# Rapid and Definitive Analysis of In Vitro DNA Methylation by Nano-electrospray Ionization Mass Spectrometry

**DOI:** 10.1007/s13361-019-02304-5

**Published:** 2019-09-16

**Authors:** Hiroshi Ushijima, Rena Maekawa, Eri Igarashi, Satoko Akashi

**Affiliations:** Graduate School of Medical Life Science, Yokohama City University, 1-7-29 Suehiro-cho, Tsurumi-ku, Yokohama, Kanagawa 230-0045 Japan

**Keywords:** CpG methylation, Nano-ESI mass spectrometry, DNA, Structural biology

## Abstract

CpG methylation of DNA is an epigenetic marker that is highly related to the regulation of transcription initiation. For analysis of CpG methylation in genomic DNA sequences, bisulfite-induced modification in combination with polymerase chain reaction (PCR) is usually utilized, but it cannot be straightforwardly applied to methylated short- and middle-sized DNAs, such as < 500 base pairs (bp), which are often utilized in structural biology studies. In the present study, we applied nano-electrospray ionization mass spectrometry (nano-ESI-MS) for the characterization of methylated DNA with < 400 bp prepared in vitro. First, double-stranded DNA oligomers were methylated with recombinant *M.Sss*I DNA methylase, which has been reported to modify completely and exclusively CpG sites in the sequence. The fragments generated by the digestion with methylation-insensitive restriction nuclease were then analyzed to identify the methylation levels by nano-ESI-MS, without liquid chromatography (LC) separation. By methylation-insensitive nuclease digestion, we divided the DNA strands into several fragments, and nano-ESI-MS enabled the accurate analysis of methylation levels in the DNA fragments with a relatively small amount of DNA sample prepared under optimized conditions. Furthermore, it was revealed that *M.Sss*I methylase hardly modifies the CpG sites closely positioned at the ends of linear DNA. The present method is similar to the strategy for post-translational modification analysis of proteins and is promising for the rapid and definitive characterization of methylated DNA that may be used in structural biology studies.

**Electronic supplementary material:**

The online version of this article (10.1007/s13361-019-02304-5) contains supplementary material, which is available to authorized users.

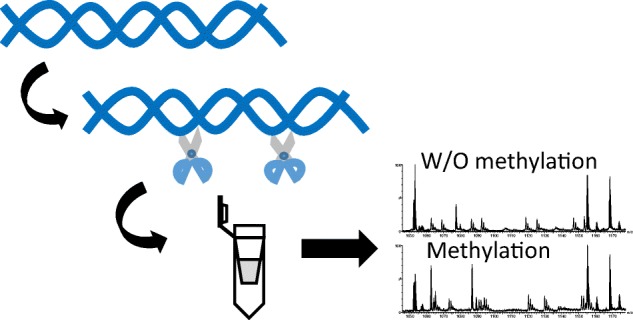

## Introduction

Transcription initiation is highly regulated by various biomolecules via epigenetic modifications of histone proteins and DNA [1–6]. Since it has been reported that disorders in epigenetic modification are related to various diseases [7–9], characterization of these modifications is being widely investigated. CpG methylation in DNA, one epigenetic marker, manages to alter the interaction of transcription factors with DNA [10, 11], usually resulting in the repression of transcription initiation [12–15]. Therefore, the characterization of CpG methylation is crucial for understanding the regulatory mechanisms of eukaryotic transcription initiation.

To unveil the mechanism of transcription regulation, genome-wide CpG methylation analyses have been performed, such as for human tissues and cells [16–18]. The most widely used method for global characterization of CpG methylation sites is DNA sequence analysis in combination with bisulfite treatment and polymerase chain reaction (PCR) [19]. This method is sophisticated and easy to perform, but it is difficult to apply in characterizing CpG methylation in relatively short, i.e., < 500 base pairs (bp), DNA fragments. Furthermore, DNA with palindromic and/or repeated sequences cannot be analyzed using this method.

For understanding of the role of epigenetic markers in transcription initiation, structural characterization at the molecular and atomic levels of modified nucleosome core particle (NCP), the minimum structural unit of chromatin, is necessary. In these structural biology studies, DNA oligomers and histone proteins, which are the NCP components, are prepared in vitro and used as parts for NCP reconstitution [20]. Following the determination of more than 100 structures of NCPs reconstituted with unmodified histone variants originating from various organisms and unmodified ~ 150 bp DNA oligomers, a couple of NCP structures reconstituted with CpG methylated DNA and unmodified histone proteins have been recently reported. Fujii et al. designed CpG146, an α-satellite-based DNA of 146 bp containing four CACGTG sequences in each strand and performed CpG methylation with *M.Sss*I DNA methylase [21]. In CpG146, eight cytosine bases in all of the CpG sequences can theoretically be methylated by *M.Sss*I methylase. Independent of the above study, Osakabe et al. reconstituted NCP with 160 bp human satellite 2 DNA containing 14 CpG sites, which were located in the TTCGAA sequence [22]. Prior to reconstitution of the NCP, the cytosine bases of all CpG sites in this DNA oligomer were methylated with *M.SssI* methylase. In these studies, the DNA sequence was mutated in such a way that all of the CpG sites were located in a single type of the sequence, CACGTG or TTCGAA. Consequently, it was possible to confirm if all CpG sites in the DNA sequences were methylated by digestion with a methylation-sensitive restriction nuclease, such as *Eco72*I (for CACGTG) or *BstB*I (for TTCGAA), in combination with gel electrophoresis. That is, *Eco72*I and *BstB*I do not cleave the phosphodiester bonds in CpG, in which the 5-position of the cytosine residue is methylated. Methylation in CpG can be identified by gel electrophoresis of the digested DNA fragments. This method is convenient, but it is applicable only for DNA oligomers that have CpG sites within a single particular xyCGzw sequence (x, y, z, w = A, T, G, or C); there is a limitation of the sequence variety of DNA in using this methylation-sensitive nuclease and gel electrophoresis method for verification of methylated sites.

Since methylation causes a 14 Da increase for each CpG site, mass spectrometry is expected as a useful method for monitoring the methylation levels in DNA fragment with < 500 bp. Particularly, matrix-assisted laser desorption ionization time-of-flight mass spectrometry (MALDI-TOFMS) may work well for characterization, because it gives a singly charged ion for each single-stranded DNA, without any complicated procedures for sample preparation [23, 24]. However, a nucleotide has a phosphate group, which is generally paired with a monovalent cation, such as Na^+^ and K^+^, in aqueous solutions, making it difficult to judge the 14 Da increase at each CpG site in a DNA fragment. In addition, it is challenging to observe intact ions of DNA oligomers by MALDI-TOFMS with high sensitivity as is the case with peptides/proteins, even in the negative ion mode. Since MALDI-TOFMS is more accurate than gel electrophoresis, it is practical to apply it for a rough estimation of the molecular mass of a DNA oligomer if a relatively large amount of sample is available for analysis [25]. However, it is not easy to precisely determine the methylation levels in DNA fragments with > 10 kDa by MALDI-TOFMS.

From the point of view of mass accuracy, ESI-MS is a more promising method for the determination of methylation levels in DNA fragments with < 500 bp. However, it requires extensive levels of sample desalting prior to MS compared with MALDI-TOFMS [26, 27]. To desalt and analyze the mass of DNA fragments, reversed phase LC-ESI-MS would be one compelling method [28], but it does not always allow rapid analysis of DNA fragments and it requires optimization of LC separation conditions depending on the DNA length. Currently, ESI mass spectrometry of PCR products has been performed mainly with Orbitrap or FT-ICR MS, because high resolution is preferable to distinguish sodium or potassium adducts in DNA by accurate mass measurement [26, 27]. It has also been reported that CpG methylation in 8-mer single-stranded DNA could be identified with ESI-triple quadrupole mass spectrometry [29].

In the present study, we have examined a method using nano-electrospray time-of-flight mass spectrometry (nano-ESI-TOF-MS) in combination with restriction-nuclease digestion that is insensitive to CpG methylation for the characterization of in vitro methylation of DNA oligomers. By ESI-TOF-MS, it is possible to absolutely reveal the methylation levels of DNA, just like the analysis of post-translational modification of proteins, on the condition that cations are excluded as much as possible. The advantages of this method are rapidity, accuracy, and applicability in analyzing the methylation levels in DNA oligomers with < 500 bp designed for structural biology research, even if they have palindromic sequences that cannot accurately be analyzed by a DNA sequencer in combination with bisulfite treatment. Methylation in any sequence can be analyzed. It should also be noted that a small amount of sample is required for mass measurement with nano-ESI-TOFMS. Since methylated DNA oligomers are prepared for NCP reconstitution subjected to structural biology study, nano-electrospray is advantageous to save the sample amount for structural analysis. We have analyzed in vitro methylated DNA oligomers with 40, 147, and 366 bp lengths and found this method effective for the definitive identification of methylation levels of DNA oligomers. Furthermore, we discovered, for the first time to our knowledge, that *M.Sss*I methylase does not modify CpG sites positioned closely to the 5′ or 3′ end in linear DNA, although it has long been believed to completely and exclusively modify the cytosine residues in CpG sequences [30, 31].

## Experimental Section

### Preparation of Methylated DNA Oligomers

**Figure 1:**
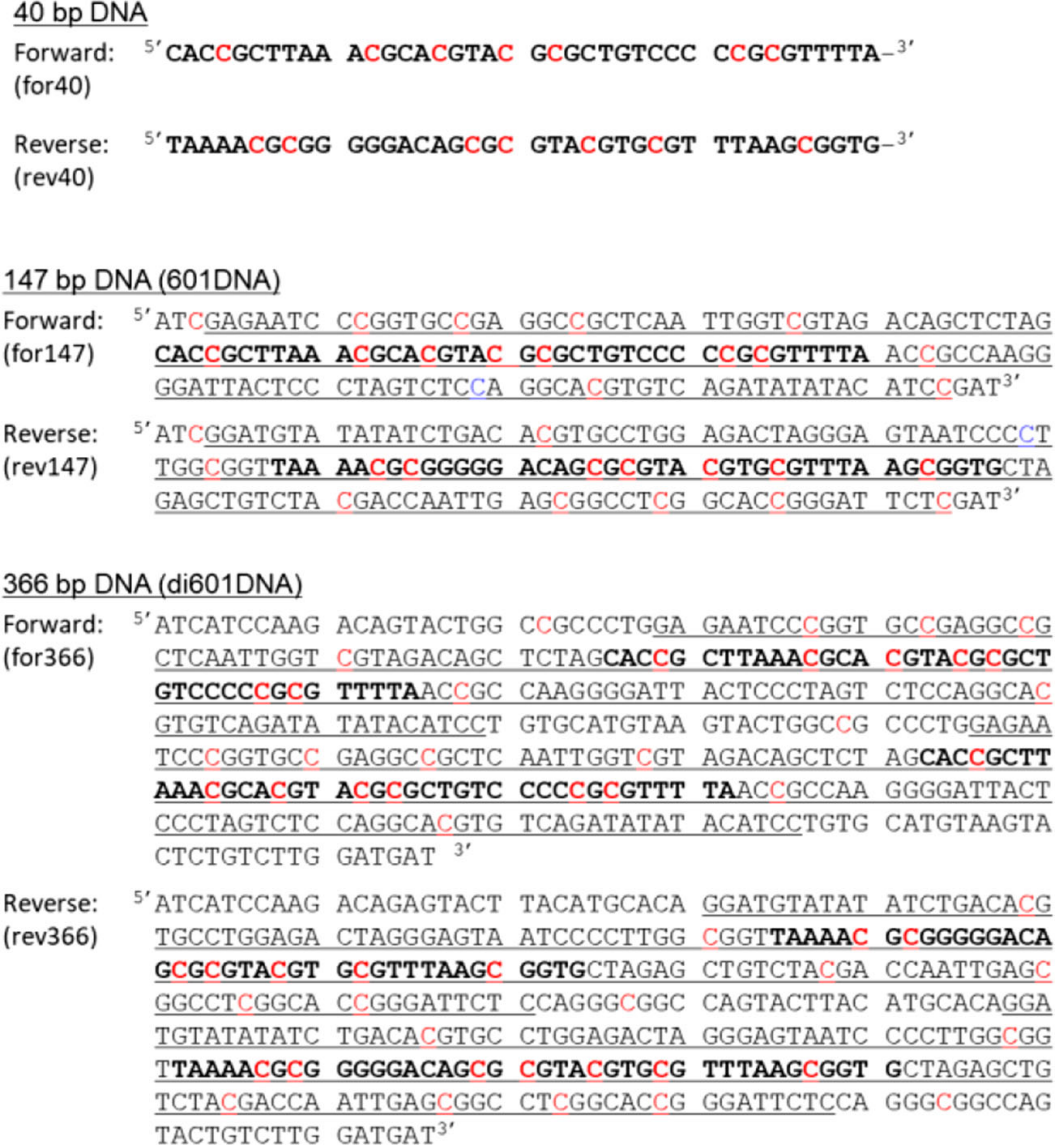
DNA sequences of 40 bp, 147 bp, and 366 bp fragments. Cytosine residues in the CpG sequence, which are possible methylation sites for *M.Sss*I methylase, are indicated in red. The second cytosine residue in the CCTGG or CCAGG, which was intrinsically methylated during cultivation of *E. coli* DH5α cells, is indicated in blue in the sequence of 147 bp DNA. Underlining corresponds to the Widom 601 DNA sequence, which is common in 147 and 366 bp DNA. Bold letters show the sequence that is identical to the sequence of the 40 bp DNA

The sequences of 40, 147, and 366 bp DNA are indicated in Figure 1. The sequence of 147 bp DNA corresponds to the Widom 601 sequence of DNA [32], whereas the 366 bp DNA has two Widom 601 sequences with 141 bp and three linker regions. It has been confirmed that the 147 bp DNA forms a mononucleosome core particle (monoNCP) [33] and the 366 bp DNA forms a dinucleosome core particle (diNCP) (unpublished) by native mass spectrometry and gel electrophoresis.

Two complementary single-stranded DNA oligomers with 40 bases, for40 and rev40, were purchased from Hokkaido System Bioscience (Japan) and used without further purification. The sequence of for40 corresponds to nucleotides 51 to 90 of the forward strand of the 147 bp DNA (for147), whereas rev40 corresponds to numbers 58 to 97 of the reverse strand of the 147 bp DNA (rev147). For making 40 bp DNA with two single-stranded DNA fragments, two DNA strands of for40 and rev40 were mixed at a concentration of 25 μM in 10 mM Tris-HCl (pH 8.0), 1 mM EDTA, and 100 mM NaCl and annealed by heating at 95 °C for 10 min, followed by slow cooling in a Styrofoam box overnight.

To prepare the 147 and 366 bp DNA with high efficiency, eight copies of 147 and 366 bp DNA fragments were inserted into pGEM-T Easy (Promega, Madison, WI) and pUC57 (GenScript, Piscataway, NJ) vectors, respectively [34]. For the preparation of 147 bp DNA, *Escherichia coli* DH5α cells were transformed with the plasmid containing eight copies of the Widom 601 sequence [34]. For the preparation of the 366 bp DNA, *E. coli* HST04 dcm−/dam− cells, which do not methylate the 5-position of cytosine in any sequence, were transformed with the plasmid containing eight copies of the fragments. The 147 and 366 bp DNA plasmids were amplified by cultivation of *E. coli* cells in SB medium, and the amplified plasmids were collected and digested with the restriction enzyme *Eco*RV. Subsequently, the 147 and 366 bp DNA were purified in a similar manner with the previous report [35]. Briefly, the 147 and 366 bp DNA were subjected to anion-exchange column chromatography (MonoQ 10/100GL, GE Bioscience, Chicago, IL) or electrophoresis (Model 491 Prep Cell, BioRad, Hercules, CA) after precipitation with polyethyleneglycol 6000 (PEG6000) and purified.

### In Vitro Methylation of the DNA Fragments

CpG methylation was performed with *M.Sss*I methylase. The pET15b plasmid containing the DNA region coding *M.Sss*I methylase was kindly provided by Dr. Takashi Umehara of RIKEN. Recombinant *M.Sss*I methylase was overexpressed in *E. coli* and purified according to the previously reported method [21]. Briefly, *M.Sss*I methylase with an N-terminal His6-tag was overexpressed in *E. coli* and purified with Ni-NTA column chromatography (Ni Sepharose 6 Fast Flow, GE Bioscience). Without cleavage of the N-terminal His6-tag, the enzyme was used for in vitro methylation.

For CpG methylation, the DNA oligomer at a concentration of 0.02 μg/μL was incubated with *M.Sss*I methylase at a ratio of 15:1 (w/w) at 37 °C for 5 h in 10 mM Tris-HCl buffer (pH 8.0) containing 50 mM NaCl, 2.5 mM EDTA, and 640 μM S-adenosylmethionine (SAM) [21]. The methylation reaction was quenched by heating the solution at 65 °C for 20 min. The methylated DNA was recovered from the solution by ethanol precipitation (described below).

### Digestion with Restriction Nuclease

To generate small DNA oligomers for accurate mass analysis, digestion with methylation-insensitive enzymes was carried out, referring to the protocol provided by the nuclease manufacturer. For 40 and 147 bp DNA, digestion with the restriction nuclease *Mse*I (New England Biolabs, Ipswich, MA), which recognizes the TTAA sequence was performed. For 366 bp DNA, a one-pot digestion with *Sca*I (New England Biolabs), which recognizes the AGTCAT sequence, and *Mse*I was carried out. The digestion was performed at 37 °C overnight in CutSmart Buffer (New England Biolabs) consisting of 20 mM Tris-acetate (pH 7.9), 50 mM potassium acetate, 10 mM magnesium acetate, and 100 μg/mL BSA.

### Purification of DNA for nano-ESI-MS

To optimize DNA purification procedures, 40 bp DNA was used for examination. After methylation and/or nuclease digestion, which were performed in involatile buffers containing inorganic salts, DNA fragments were isolated from the solution by ethanol precipitation. To optimize the experimental conditions, the DNA sample was treated with the methylation reaction solution and that for nuclease digestion, without enzymes, prior to ethanol precipitation. Three protocols of ethanol precipitation and desalting procedures were examined for unmethylated 40 bp DNA. The DNA fragments precipitated with ethanol in the presence of 3 M sodium acetate were dissolved in 50 mM triethylammonium acetate (TEAA) (pH 7.0), and then desalted by size exclusion with a BioRad P-6 spin column equilibrated with 50 mM TEAA (Protocol I). Alternatively, the DNA fragments precipitated with ethanol in the presence of 7 M ammonium acetate were dissolved in MilliQ water (Protocol II) or 50 mM ammonium acetate (Protocol III), and then desalted with a BioRad P-6 spin column equilibrated with MilliQ water (Protocol II) or 20 mM ammonium acetate (Protocol III). For nano-ESI-MS, the DNA sample in 50 mM TEAA (Protocol I) was diluted with acetonitrile at a ratio of 1:1 (v/v). In the case of the DNA sample prepared with MilliQ water (Protocol II), it was diluted with acetonitrile containing 2 M triethylamine (TEA) at a ratio of 1:1 (v/v), to let the double-stranded DNA dissociate, and then it was subjected to nano-ESI-MS. The DNA sample prepared with ammonium acetate (Protocol III) was diluted with methanol at a ratio of 1:1 (v/v) and subjected to nano-ESI-MS.

### Nano-ESI-MS

Nano-ESI mass spectra were obtained with a SYNAPT G2 HDMS mass spectrometer (Waters, Milford, MA) with a nano-ESI source [33, 36] in the negative ion mode. The concentration of DNA was estimated with UV absorption at 260 nm. To obtain nano-ESI mass spectra, an aliquot of 4 μL of the sample solution containing 0.3–4 μM DNA was deposited in a nano-spray tip with an i.d. of 5 μm (HUMANIX, Japan), and then positioned in the nano-ESI source. After optimization of the measurement conditions, the following parameters were applied for observation of DNA ions: 0.7–0.8 kV capillary voltage, 20 V sampling cone voltage, and 4 V trap collision energy. The quadrupole was operated in the automatically scanning mode. Mass calibration was performed with (CsI)_n_I^−^ ion clusters in the range of *m/z* 500–4000, using 2 mg/mL CsI in 50% isopropyl alcohol. Mass spectra were acquired by accumulation for 2 min and processed with MassLynx 4.1 software, and experimental masses of DNA fragments were obtained using the most intense isotope peaks.

## Results and Discussion

### Optimization of Preparation Protocols for ESI-MS of Unmethylated DNA

**Figure 2:**
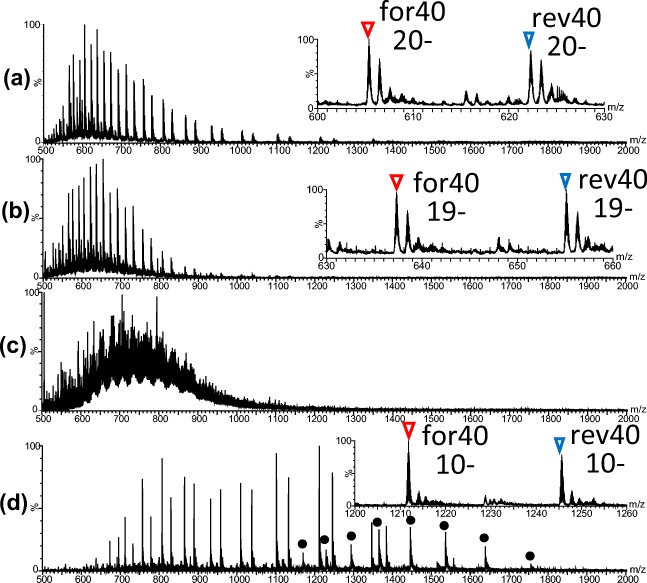
Nano-ESI mass spectra of unmethylated DNA. (**a**) Nano-ESI mass spectrum of the mixture of for40 and rev40. (**b**) Nano-ESI mass spectrum of 40 bp DNA prepared by protocol II: ethanol precipitation in the presence of 7 M ammonium acetate, solubilization with MilliQ water, and desalt with a P-6 spin column equilibrated with MilliQ water. The desalted sample was mixed with 2 M TEA in acetonitrile at a ratio of 1:1. (**c**) Nano-ESI mass spectrum of the *Mse*I digest of 147 bp DNA prepared by protocol II. (**d**) Nano-ESI mass spectrum of 40 bp DNA prepared by protocol III: ethanol precipitation in the presence of 7 M ammonium acetate, solubilization with 50 mM ammonium acetate, and desalt with a P-6 spin column equilibrated with 20 mM ammonium acetate. The desalted sample was mixed with methanol at a ratio of 1:1. Insets in (**a**), (**b**), and (**d**) indicate expanded mass spectra, showing the most intense peaks. Red and blue reversed triangles correspond to for40 and rev40, respectively. Black closed circles in (d) indicate peaks of double-stranded 40 bp DNA

To unambiguously analyze the CpG methylation levels in DNA fragments using mass spectrometry, the involatile monovalent cations in the sample solution must be decreased as much as possible. Figure 2a, b, and d show ESI mass spectra of the unmethylated 40 bp DNA prepared by different procedures. Figure 2a shows the ESI mass spectrum of the two synthetic 40-base DNA fragments mixed and subjected to ESI-MS without annealing. The sample was prepared in 20 mM TEAA (pH 7.0) with 50% acetonitrile, and sharp peaks were found at *m/z* 500–1400. Peaks for each DNA strand were observed in a wide range of charged states, despite the sample solution being neutral at pH 7.0. A few adduct monovalent cations were recognized for the DNA peaks (Figure 2a, inset). For methylation with *M.Sss*I in vitro, double-stranded DNA should be prepared. Thus, these two 40-base fragments were annealed and subjected to nano-ESI-MS, for which the sample was prepared by Protocol I. For nano-ESI-MS, the desalted sample was mixed with acetonitrile at a ratio of 1:1 (v/v). That is, the solution for nano-ESI-MS was identical to that for the mixture of for40 and rev40. Ions of the double-stranded 40 bp DNA peaks were observed at *m/z* 1400–2750 with high intensity in the mass spectrum, but single-stranded 40-base DNA peaks were less observable at *m/z* 700–1500 (Supplementary Figure S-1). To discuss the methylation levels, it is preferable to obtain the accurate molecular mass of the single-stranded DNA. Thus, sample preparation methods were re-examined. The sample was prepared according to Protocol II. For nano-ESI-MS, the desalted sample was mixed with 2 M TEA in acetonitrile at a ratio of 1:1. In the ESI mass spectrum of 40 bp DNA prepared by Protocol II, the peaks of two single-stranded DNA oligomers were observed at *m/z* 500–1000 associated with a few monovalent cations (Figure 2b). Peaks of single strands shifted to the lower *m/z* region compared with those in the spectrum in Figure 2a because of the high pH of the sample solution containing a high concentration of TEA. When Protocol II was applied to the *Mse*I digest of unmethylated 147 bp DNA, however, DNA peaks were associated with many sodium ions and it was difficult to assign them to the DNA fragments (Figure 2c). Consequently, the preparation method was further optimized. Next was Protocol III; ethanol precipitation in the presence of 7 M ammonium acetate, solubilization with 50 mM ammonium acetate, and desalting with a P-6 column equilibrated with 20 mM ammonium acetate were applied to the 40 bp DNA sample. Since ammonium acetate, which is a common solvent for native MS of structured DNA, was used for the P-6 column elution, methanol was added to the solution at a ratio of 1:1 (v/v) for nano-ESI-MS to disrupt hydrogen bonds within the double-stranded DNA. As shown in Figure 2d, peaks of the single-stranded DNA were clearly observed at *m/z* 500–1400, which were associated with double-stranded DNA peaks at higher *m/z* values. Few sodium and potassium adducts to the single-stranded DNA peaks were recognized, as shown in the inset of Figure 2d. Accordingly, for further nano-ESI-MS experiments, DNA samples with or without methylation were prepared according to Protocol III and diluted with methanol for nano-ESI-MS.

### ESI-MS of Methylated 40 bp DNA

**Figure 3:**
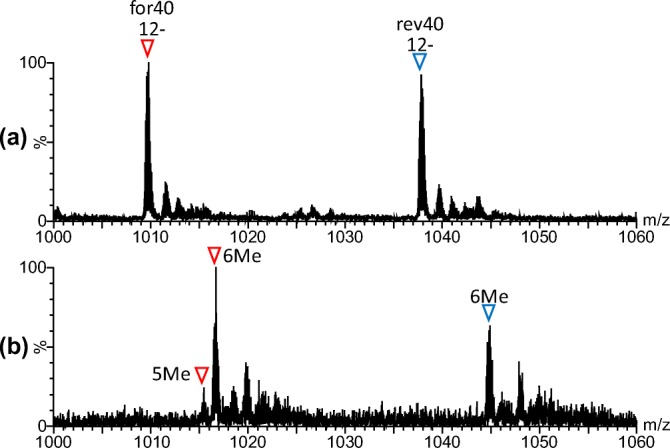
Nano-ESI mass spectra of unmethylated (**a**) and methylated (**b**) 40 bp DNA. Expanded mass spectra for *m/z* 1000–1060 are indicated. Red and blue reversed triangles indicate signals of for40 and rev40, respectively. Experimental masses of the observed peaks are summarized in Table 1. Wide-range mass spectrum (*m/z* 500–2000) of methylated 40 bp DNA is indicated in Supplementary Figure S-2

In the ESI mass spectra of 40 bp DNA with and without methylation, multiply charged ions of the dissociated two single DNA strands, for40 and rev40, were principally observed (expanded for *m/z* 1000–1060: Figure 3a, b; wide range (*m/z* 500–2000) (unmethylated): Figure 2d; wide range (*m/z* 500–2000) (methylated): Supplementary Figure S-2). Theoretical and observed mass values of the fragments are summarized in Table 1. From the observed mass values, it was suggested that six cytosine residues among seven CpG sites in for40 and rev40 were methylated. For for40, five methylated ions were also observed with low intensity. No experimental condition was found for complete methylation of the 40 bp DNA fragment. This result is contrary to the previous report that *M.Sss*I methyltransferase completely and exclusively methylates the cytosine residues in all CpG sites [30, 31]. To identify which cytosine residue was not methylated, digestion with restriction nuclease *Mse*I, which recognizes the TTAA sequence, was performed on methylated 40 bp DNA and each DNA fragment was divided into two fragments. The digest was then subjected to nano-ESI-TOF-MS (Supplementary Figure S-3 and Table 1). In the mass spectrum, peaks corresponding to 1F_40 (from 1st to 7th) and 2F_40 (from 8th to 40th) of for40 and 1R_40 (from 32nd to 40th) and 2R_40 (from 1st to 31st) of rev40 were observed. The experimental mass of each fragment is summarized in Table 1.

**Table 1:**
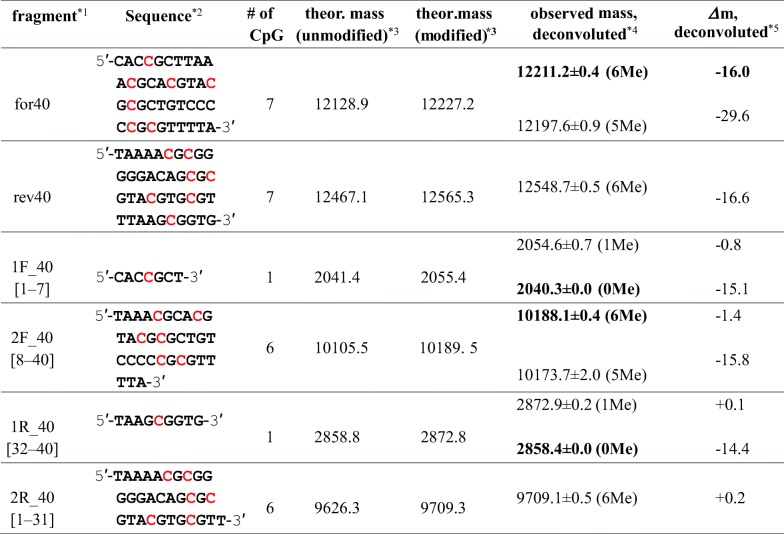
Theoretical and Experimental Masses of 40 bp DNA Fragments Obtained by Digestion with *Mse*I

*^1^The number in square brackets shows the position of the fragment in the sequence of 40for or 40rev

*^2^C in red shows a possible methylation site in the CpG sequence

*^3^Average mass

*^4^Centered mass of the most intense peak among each isotope envelope is indicated. Experimental masses of the main observable species are indicated with bold letters. The number of methylated CpG regions is indicated in parenthesis

*^5^Difference between the theoretical (expected maximum level of methylation) and observed masses is indicated

It was identified that the principal species for 1F_40 and 1R_40 were not methylated, and that minor species with one methylation also existed for these short fragments. MS/MS experiment of the minor species was unsuccessful due to low ion intensity. In 1F_40 and 1R_40, only one CpG sequence was included at regions 4–5 of for40 and 36–37 of rev40. Thus, it was found that the cytosine residues at the 4th and 36th positions in for40 and rev40, respectively, located close to the 5′ or 3′ end of the DNA fragment, were slightly methylated with *M.Sss*I methylase. In contrast, the fragment of 2R_40 was completely methylated; six methylations were identified in this region. The fragment of 2F_40 exhibited six strong methylations but weak signals of five methylated species were also observed. The fragment of 2F_40 is > 10 kDa, which is too large to analyze the sequence with collision-induced dissociation (CID) tandem mass spectrometry. In addition, peak intensity of the five methylated fragment was low. Therefore, it was impossible to definitely locate the unmethylated cytosine residue among six CpG sites. Upon analysis of these results, methylation in 40 bp DNA was easily characterized by nano-ESI-MS, and it was indicated that *M.Sss*I methylase modifies cytosine residues in the CpG sites located in the middle of the sequence but hardly methylates cytosine residues closely positioned to the 5′ or 3′ end of the linear DNA strands.

### ESI-MS of Methylated 147 bp DNA

**Figure 4:**
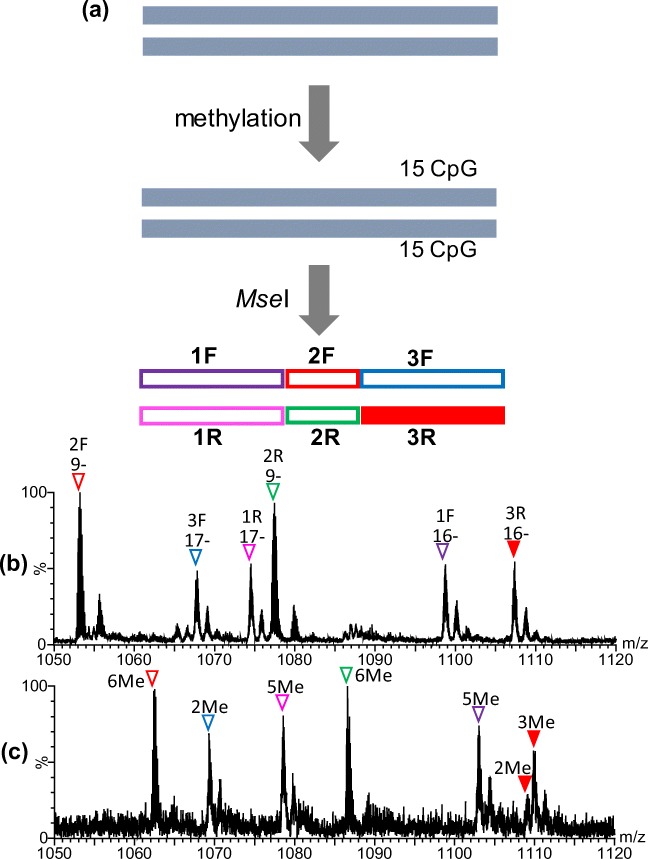
Experimental scheme of methylation analysis of 147 bp DNA (**a**) and nano-ESI mass spectra obtained for147 bp DNA without (**b**) and with (**c**) methylation. Expanded mass spectra for *m/z* 1050–1120 are indicated. Colored reversed triangles associated with the labels on peaks, such as 1F and 1R, as well as charge states, show the ions of the DNA fragments originating from the 147 bp DNA. The position and experimental mass of each fragment are summarized in Table 2. Full-range mass spectra are indicated in Supplementary Figure S-4

Since 147 bp DNA was amplified by cultivation of *E. coli* DH5α cells, the second cytosine residue in the sequence of CCXGG (X = A or T) was methylated by an intrinsic methylase. Thus, the theoretical molecular masses of for147 and rev147, both of which have a single CCXGG sequence, were calculated as 45,122.2 and 45,593.5, respectively, before CpG methylation. In the sequences of for147 and rev147, there are 15 CpG sites (Figure 1), and it is not easy to analyze the number of modified cytosine residues in long intact DNA strands with > 40 kDa. To accurately determine the number of methylated cytosine residues, therefore, the 147 bp DNA with or without methylation was subjected to digestion with a restriction nuclease, *Mse*I, prior to nano-ESI-TOF-MS. Digestion with *Mse*I generated three DNA oligomers from each single-stranded DNA, 1F_147, 2F_147, and 3F_147 from for147, and 1R_147, 2R_147, and 3R_147 from rev147 (Figure 4a and Table 2).

**Table 2:**
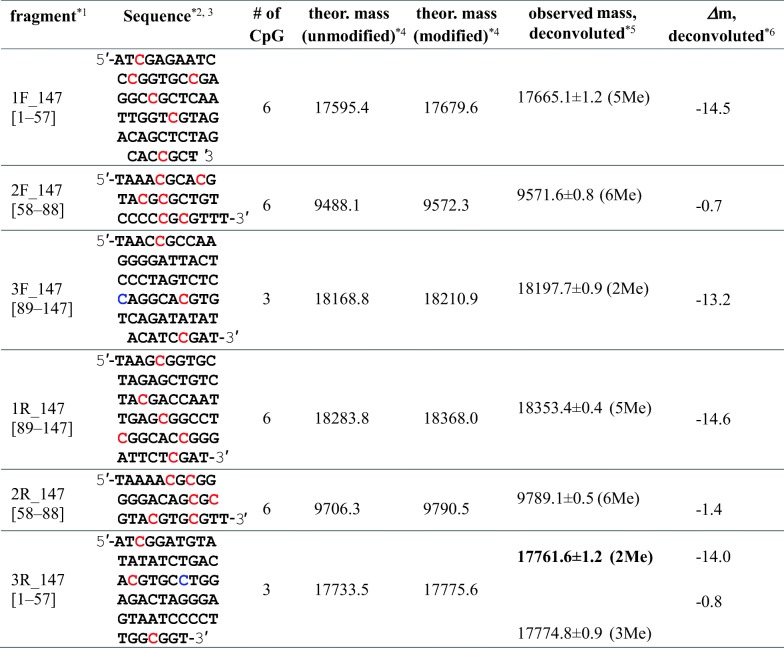
Theoretical and Experimental Masses of DNA Fragments Obtained by Digestion of 147 bp DNA with *Mse*I

*^1^The number in square brackets shows the position of the fragment in the sequence of 147for or 147rev

*^2^C in red shows a possible methylation site in the CpG sequence

*^3^Since 147 bp DNA was prepared in *E. coli* DH5α cells, the C-5 position of the second cytosine residues in the sequences of CCAGG and CCTGG, indicated in blue, was intrinsically methylated

*^4^Average mass

*^5^Centered mass of the most intense peak among each isotope envelope (resolved) or centered mass of the observed unresolved isotope envelope is indicated. Experimental masses of the main observable species are indicated with bold letters. The number of methylated CpG regions is indicated in parenthesis

*^6^Difference between the theoretical (expected maximum level of methylation) and observed masses is indicated

As shown in Figure 4b, c and Supplementary Figure S-4, signals for six DNA fragments, as expected, could clearly be observed in a single mass spectrum. The experimentally obtained mass of each methylated DNA fragment is summarized in Table 2. By comparing the experimental masses of fragments with and without methylation, we found that all six of the cytosine residues were methylated in 2F_ and 2R_147 but one cytosine residue was not methylated at all in 1F_, 3F_, and 1R_147. In 3R_147, one among three cytosine residues was partly methylated. It should be noted that all of 1F_, 3F_, 1R_, and 3R_147 have CpG sequences at the 3rd or 4th position from the 5′ or 3′ end of the intact DNA with 147 bases.

The sizes of the digested DNA fragments, 1F_, 3F_, 1R_, and 3R_147, in which a single cytosine residue was almost unmodified, were ~ 18 kDa, and it was difficult to characterize them by CID experiments. Thus, the unmethylated cytosine sites in 147 bp DNA could not be definitely identified. However, considering that cytosine residues positioned 4th from the 5′ end of for40 and 5th from the 3′ end of rev40 were little modified in 40 bp DNA, as mentioned above, it was likely that *M.Sss*I does not methylate cytosine residues closely located to the end of 147 bp DNA, such as the 3rd or 4th position from the end. This is discussed later.

### ESI-MS of Methylated 366 bp DNA

**Figure 5:**
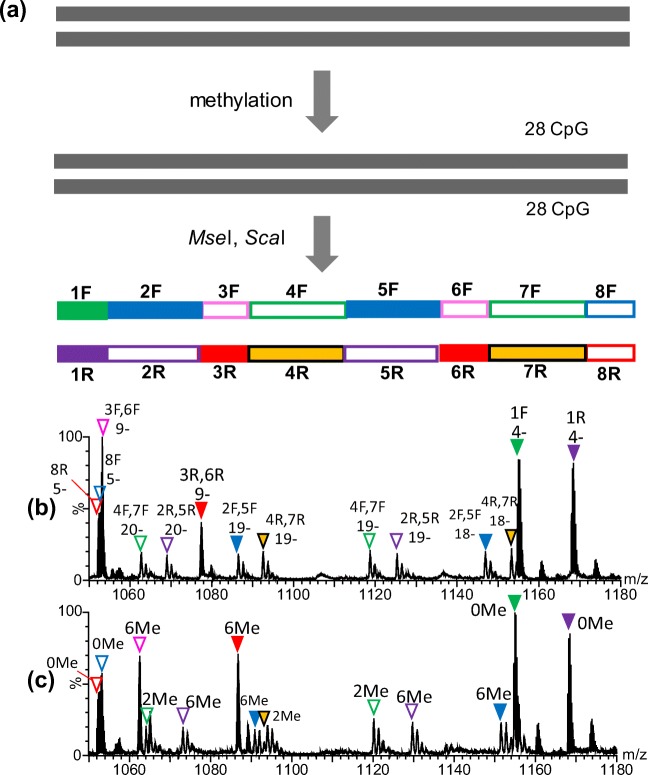
Experimental scheme of methylation analysis of 366 bp DNA (**a**) and nano-ESI mass spectra obtained for 366 bp DNA without (**b**) and with (**c**) methylation. Expanded mass spectra for *m/z* 1050–1180 are indicated. Colored reversed triangles associated with the labels on peaks, such as 1F and 1R, as well as charge states, show the ions of the DNA fragments originating from 366 bp DNA. The position and experimental mass of each fragment are summarized in Table 3. Full-range mass spectra are indicated in Supplementary Figure S-5

Methylation of the 366 bp DNA with *M.Sss*I was characterized in a similar manner to the 147 bp DNA fragment. Each of the two complementary DNA strands with 366 bases, for366 and rev366, has 28 CpG sequences (Figure 1). After methylation of the 366 bp DNA, it was subjected to digestion with two restriction enzymes, *Mse*I and *Sca*I, simultaneously. These two nucleases can cleave double-stranded DNA in the same buffer, CutSmart Buffer, and generate eight fragments from each single-stranded DNA, for366 and rev366 (Figure 5a). Table 3 summarizes the experimentally obtained masses of the methylated fragments, also showing the theoretical mass of unmodified and modified ones. Since 366 bp DNA has two sets of 141 bp from the Widom 601 sequence, six pairs of the fragments [2F and 5F, 3F and 6F, 4F and 7F, 2R and 5R, 3R and 6R, and 4R and 7R] are expected to have identical DNA sequences and theoretical masses. Figure 5b and c show expanded ESI mass spectra of the digested 366 bp DNA before and after methylation. Their full-range mass spectra are indicated in Supplementary Figure S-5. Among the fragments listed in Table 3, the theoretical masses of 8F_and 8R_366 are very close: 5271.4 and 5267.4. However, the mass resolution of SYNAPT G2 HDMS was high enough to distinguish these two fragments in the spectra (Supplementary Figure S-6). Consequently, multiply charged ions of all ten fragments could be identified in a single mass spectrum.

**Table 3:**
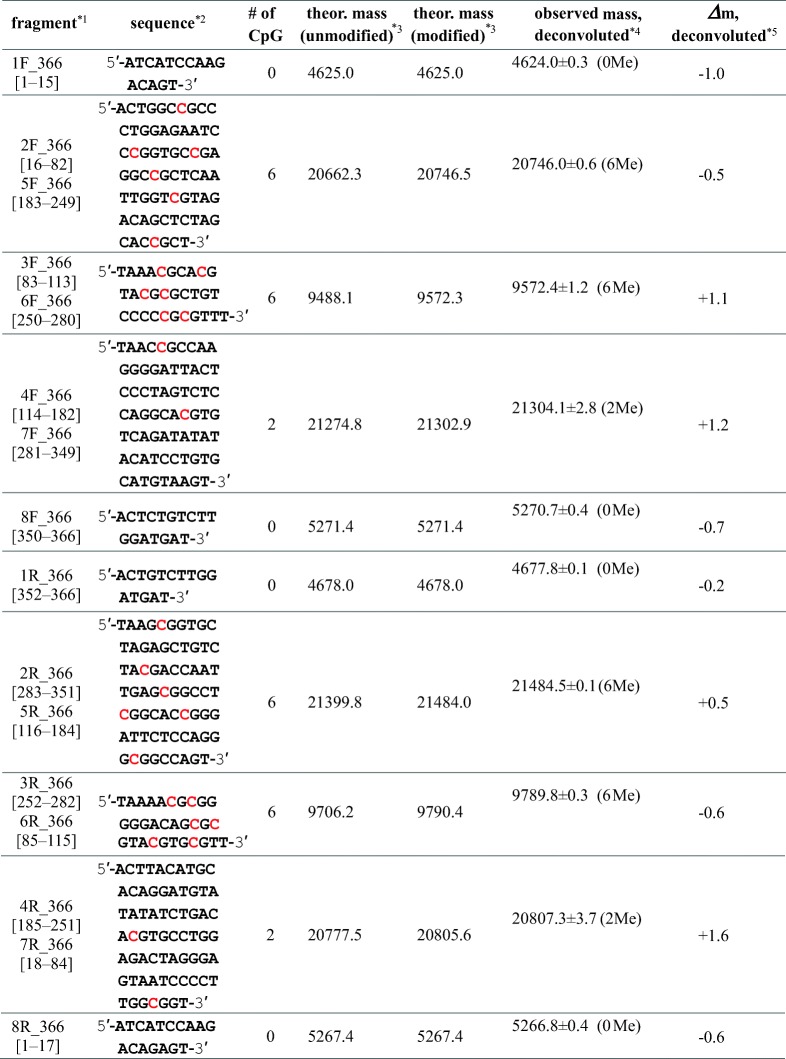
Theoretical and Experimental Masses of DNA Fragments Obtained by Digestion of 366 bp DNA with *Mse*I and *Sca*I

*^1^The number in square brackets shows the position of the fragment in the sequence of 366for or 366rev

*^2^C in red shows a possible methylation site in the CpG sequence

*^3^Average mass

*^4^Centered mass of the most intense peak among each isotope envelope (resolved) or centered mass of the observed unresolved isotope envelope is indicated. The number of methylated CpG regions is indicated in parenthesis

*^5^Difference between the theoretical (expected maximum level of methylation) and observed masses is indicated

In fragments of 1F_, 8F_, 1R_, and 8R_366, there is no CpG sequence. Therefore, no mass increase was observed after *M.Sss*I methylation, as expected. In contrast, observed masses for the other fragments, 2F_, 3F_, 4F_, 5F_, 6F_, and 7F_, and 2R_, 3R_, 4R_, 5R_, 6R_, and 7R_366 suggest that methylation had been completely performed. Consequently, as summarized in Table 3, all cytosine residues in the 56 CpG sites in 366 bp DNA were found to be completely methylated.

### *Specificity of M.Sss*I *methylase*

As mentioned above, all CpG sites in the 366 bp DNA were methylated, but a few CpG sites remained unmethylated in 40 bp and 147 bp DNA. The sequence of the 40 bp DNA is identical to those of the regions of 76–115 and 243–282 of the 366 bp DNA, and to that of the region of 51–90 of the 147 bp DNA. Since the C-5 position of every CpG site in the 366 DNA was methylated, the lack of methylation of a few CpG sites in 40 bp and 147 bp DNA would not be due to the sequence specificity of the enzyme but to the position of the cytosine residue in the sequence.

In the 40 bp DNA sequence, unmethylated cytosine residues were located at the 4th position of for40 and 5th of rev40. In the fragment 2F_40, which has six CpG sites including a cytosine residue at the 7th position from the 3′ end, six obvious and five slightly observable methylations were recognized. In the case of 147 bp DNA, unmethylated cytosine is included in the 1F_, 3F_, 1R_, and 3R_147 fragments. In contrast, there is no CpG sequence in the fragments 1F_, 8F_, 1R_, and 8R_366, which are positioned at the “end” of the long strands, and all CpG sites were methylated in the 366 bp DNA. Considering this all, *M.Sss*I methylase has difficulty in modifying the C-5 position of the CpG sequence located close to the 5′ or 3′ end in linear DNA.

As mentioned above, it was identified that *M.Sss*I methylase does not always modify the C-5 position of the CpG sequence. When *M.Sss*I methylase was first isolated and its specificity was characterized, methylation on the *Spiroplasma* sp. strain DNA, *E. coli* chromosome, and ϕX174 RF was carried out and no unmethylated cytosines in CpG sites were found [30, 31]. In these studies, characterization of enzymatic activity was carried out on circular DNA. In contrast, linear DNA was used for examination in the present study. Circular DNA strands can provide a scaffold for stably interacting with *M.SssI*, but end regions of the linear double-stranded DNA cannot interact with the methylase securely. Therefore, a few cystosine residues positioned closely to the 5′ or 3′ ends remained unmethylated.

## Conclusions

We have established a rapid and unambiguous characterization method to analyze the methylation levels of < 400 bp DNA sequences using nano-ESI-TOFMS in combination with a single-step digestion with restriction nucleases. Although no chromatographic separation of DNA fragments was performed, multiply charged ions of the digested DNA fragments were observed in a single mass spectrum. This was achieved by optimization of the sample preparation procedure of the DNA fragments for nano-ESI-MS, which enabled the analysis of DNA fragments with a relatively small amount of sample (few picomoles). In the structural biology studies, a mg level of the sample is generally prepared, but a small sample consumption for verification of the methylation levels is favorable to keep as much of the methylated DNA as possible for preparation of protein-methylated DNA complexes, such as mono- and diNCP. It should also be noted that this method, without MS/MS, does not enable explicit identification of the methylated sites, but it is quite advantageous for rapidly and definitively identifying the in vitro methylation levels of DNA fragments designed for structural biology studies.

Since mutation of the DNA sequence is not required by this method to match the specificity of restriction nucleases [21, 22], DNA with a variety of sequences can be subjected to reconstitution of protein-methylated DNA complexes for structural studies at the atomic level. In view of the fact that chromatin is composed of nucleosomes connected with linker DNA, repeats of a particular DNA sequence such as Widom 601 DNA are generally used for the in vitro preparation of nucleosomes subjected to structural biology study. Consequently, it would be possible to apply this method to confirm the methylation levels of longer DNA fragments designed for reconstitution of multiple NCP such as those with multiple DNA linkers (e.g., tri- and tetra-linked, and so forth). It would be advisable to use mass spectrometers with higher mass resolution, such as Orbitrap or FT-ICR-MS, but it is expected that TOF-MS can provide sufficient information on the methylation degree even for longer DNA fragments. Considering all, this sequence-independent method may contribute to structural biology studies for the elucidation of the structural basis of epigenetic control mechanisms by DNA methylation.

The present study demonstrated for the first time that *M.Sss*I methylase does not modify the C-5 position of the CpG sequence that is located close to the ends of linear DNA fragments, although it has long been believed to completely and exclusively modify the cytosine residues in CpG sequences [30, 31]. This could be achieved by accurate determination of the numbers of methylated cytosine residues in the CpG sequences utilizing nano-ESI-TOF-MS with optimized sample preparation procedures.

## Electronic Supplementary Material

ESM 1 (DOCX 2087 kb)
