# Supplementary material for: Rapid and Definitive Analysis of In Vitro DNA Methylation by Nano-electrospray Ionization Mass Spectrometry
Source: J Am Soc Mass Spectrom. 2019 Sep 16;30(11):2335–46. doi: 10.1007/s13361-019-02304-5 (PMC6828984; doi:10.1007/s13361-019-02304-5)
Supplement: Supplementary file 1 — (DOCX 2087 kb) [file 13361_2019_2304_MOESM1_ESM.docx]

**Rapid and Definitive Analysis of *In Vitro* DNA Methylation**

**by Nano-electrospray Ionization Mass Spectrometry**

Hiroshi Ushijima, Rena Maekawa, Eri Igarashi, and Satoko Akashi*

Graduate School of Medical Life Science, Yokohama City University, 1-7-29 Suehiro-cho, Tsurumi-ku, Yokohama, Kanagawa 230-0045, Japan

**Supporting information**

**Contents**

Supplementary Figures S-1, S-2, S-3, S-4, S-5 and S-6

**Supplementary Figures**

**Figure S-1.** NanoESI mass spectrum of 40 bp DNA prepared by Protocol I: it was precipitated with ethanol in the presence of 3 M sodium acetate, dissolved in 50 mM TEAA (pH 7.0), and desalted with a P-6 spin column equilibrated with 20 mM TEAA. For nanoESI-MS, the sample was mixed with acetonitrile at a ratio of 1:1. Black closed circles indicate peaks of double-stranded 40 bp DNA. Inset indicates the expanded mass spectrum of the most intense observed ions of single-stranded DNA, for40 and rev40. Red and blue reversed triangles correspond to for40 and rev40, respectively.


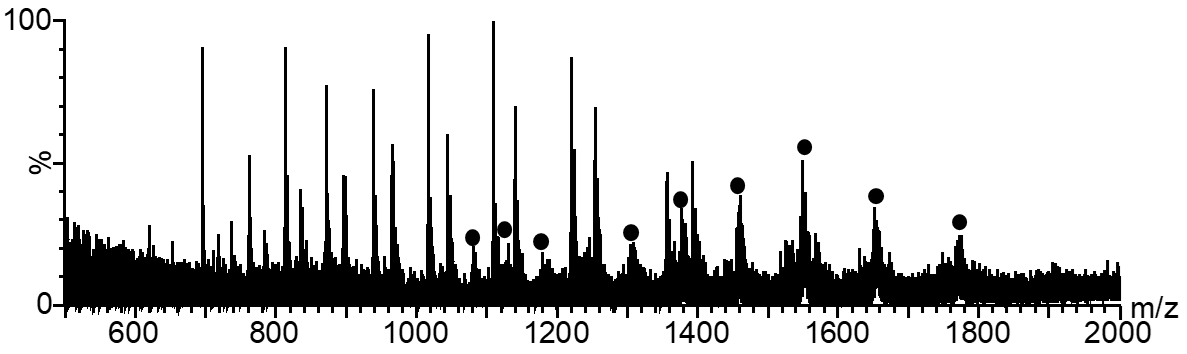


**Figure S-2.** NanoESI mass spectrum of methylated 40 bp DNA. Black closed circles correspond to peaks of double-stranded 40 bp DNA.

(a)

(b)

(c)

**Figure S-3.** NanoESI mass spectrum of *Mse*I digest of methylated 40 bp DNA. Wide range mass spectrum (*m/z* 500−2000) (a) and expanded mass spectra for *m/z* 675−725 (b) and *m/z* 840−890 (c). Ions of 1F_40 with 3- charges and 1R_40 with 4- charges, marked with green and purple reversed triangles, were observed in (b) whereas ions of 2F_40 with 12- charges and 2R_40 with 11- charges, marked with red and blue reversed triangles, were observed in (c).

(a)

(b)

**Figure S-4.** NanoESI mass spectrum of *Mse*I digest of ummethylated (a) and methylated (b) 147 bp DNA.

(a)

(b)

**Figure S-5.** NanoESI mass spectrum of *Mse*I and *Sca*I digest of methylated (a) and unmethylated (b) 366 bp DNA.

(a)

(b)

**Figure S-6.** Expanded nanoESI mass spectrum of *Mse*I and *Sca*I digest of unmethylated (a) and

methylated (b) 366 bp DNA. Reversed triangles show the monoisotopic peaks.
